# North London Hospital for Consumption

**Published:** 1893-07-15

**Authors:** 


### NORTH LONDON HOSPITAL FOR CONSUMPTION.

An interesting ceremony took place at Hampstead on July 4th, when the new central block was formally opened by Her Royal Highness Princess Christian. There were present, amongst others, the Bishop of Marlborough, Lord Robartes, Lord Knutsford, the Rev. S. B. Burnaby and Mrs. Burnaby, he Rev. G. A. Huklots and Mrs. Huklots, the Rev. Dr. Newman Hall and Mrs. Hall, Dr. Bernard O'Connor, Major-General R. Young, and Colonel A. M. Arthur. Princess Christian, who was accompanied by Princess Victoria of Schleswig-Holstein, reached the hospital about four o'clock, and was conducted to the daïs in the marquee, where H.R.H. was welcomed by Lord Knutsford, who afterwards read an address. The Princess having declared the block open, purses were presented to her by a number of ladies and children in aid of the Building Fund; after which the Bishop of Marlborough conducted a short service. Before leaving Princess Christian inspected some of the wards. The central block is an important addition to the present building, and provides accommodation for thirty-five extra patients, bringing the total number of beds up to eighty, and extra room, which was greatly needed, is also given to the nursing staff. To complete the original design another wing has yet to be added, and it is much to be hoped that funds will soon be forthcoming for that purpose.

